# LEVELING UP ADHERENCE PREDICTION: MOTIVATION’S INFLUENCE ON ENGAGEMENT WITH TECH-BASED COGNITIVE INTERVENTIONS

**DOI:** 10.1093/geroni/igae098.3553

**Published:** 2024-12-31

**Authors:** Rinanda Shaleha, Nelson Roque, Erin Harrell

**Affiliations:** The Pennsylvania State University, University Park, Pennsylvania, United States; The Pennsylvania State University, University Park, Pennsylvania, United States; National Institute on Aging, Baltimore, Maryland, United States

## Abstract

In this study, comprising a sample size of N=118 older adults with an age range of 65 years and above, we investigated the impact of individual motivation on adherence behavior within a technology-based cognitive training intervention. Despite previous research indicating limited effects of framing conditions (negative, positive, and disabled) on adherence (Harrell et al., 2021), we hypothesized that individual motivation levels would significantly influence adherence behavior. Motivation is operationally defined based on participants’ performance in cognitive training games, where high motivation is indicated by a pattern of low failure rates and successful game performance. Utilizing advanced multilevel modeling techniques, we compared adherence models with and without motivation as a predictor. Our findings supported our hypothesis. The linear mixed model analysis revealed a significant effect of motivation on adherence (Estimate = 1.35939, SE = 0.24490, t (1422.78) = 5.551, p <.001), indicating that for each unit increase in motivation, there is a significant increase in adherence. Likelihood ratio tests (LRTs) comparing the model fit showed significantly divergent log-likelihoods between the two models (Δχ² (1) = 27.559, p <.001). These findings suggest that high levels of motivation play a crucial role in driving adherence behavior within the context of technology-based cognitive training interventions. By leveraging motivational factors and harnessing the power of gamification, researchers and practitioners can design more effective interventions that promote adherence, facilitate behavior change, and ultimately improve outcomes for participants.

